# In-situ fabrication of Cr doped FeNi LDH on commercial stainless steel for oxygen evolution reaction

**DOI:** 10.1038/s41598-023-50361-4

**Published:** 2024-01-09

**Authors:** Yanhong Lv, Xinrong Deng, Jingjing Ding, Yang Zhou

**Affiliations:** 1 School of Physical and Chemistry, Hunan First Normal University, Changsha, 410205 Hunan China; 2 College of Chemistry and Chemical Engineering, Hunan University, Changsha, 410082 Hunan China

**Keywords:** Energy science and technology, Materials science, Electrocatalysis

## Abstract

Commercial stainless steel has attracted increasing interest due to their rich content in transition metal elements and corrosion resistance properties. In this work, we design a facile and rapid route to in-situ fabricate the Cr doped FeNi layered double hydroxides nanosheets (LDHs) on modified stainless steel (Cr–FeNi LDH @ ESS) under ambient condition.The ultra small scaled 2D structure only around 20 nm diameter and metal ions with multivalent oxidation state were observed on the in situ fabricated LDHs, which provides high active area and active sites and thus promote excellent oxygen evolution reaction (OER). The Cr–FeNi LDH @ESS electrocatalysts exhibit an over potential of 280 mV at 10 mA cm^−2^ and achieves a Tafel slope of 44 mV dec^−1^ for OER in the 1.0 M KOH aqueous solution. We anticipate that the operating strategy of our system may promote the development of commercial non-precious productions as the efficient electrocatalysts for energy storage and conversion.

## Introduction

Electrochemical water splitting is a most of promising friendly environmental technologies for producing high purity hydrogen and has attained considerable attentions^1–3^. The intrinsically kinetic energy of two half reactions involved of oxygen evolution reaction (OER) and hydrogen evolution reaction (HER) has a great effect on the reaction efficiency. Especially, OER with a complicated four electron and proton transfer process usually has sluggish kinetics and high OER overpotential, so that the OER anode limits the efficiency of the whole water splitting and the operating cell voltage is much larger than the theoretical voltage (1.23 V)^4^. Although the noble metals compounds, such as RuO_2_, IrO_2_, show comparatively low overpotential for OER, their high cost and scarce storage in the Earth limit the large-scale production and application in the water splitting^5,6^. Thus, it is necessary to design and synthesize highly efficient, stable and non-precious electrocatalysts.

Many studies have been devoted to develop low cost and highly active electrocatalysts for OER based on the transition mental based compound (TMs)^7–10^. Among them, metal layered double hydroxides (LDHs) and oxyhydroxides have been widely reported due to their special two-dimensional structure, ion intercalation ability and considerable catalytic activity for OER^11–16^. Compared to other bimetallic LDHs, FeNi based LDHs is considered as promising OER electrocatalysts because of their low kinetic barriers in the rate-determining step as effective OER electrocatalysts in alkaline solutions^17–21^. Thus, many researches were devoted to improve the electrocatalytic activity of FeNi based LDHs^22–27^. Enhancing the activity surface area and modifying the electronic structure of active sites are effective strategy for improving the electrocatalytic activity of FeNi based LDHs for OER^28–31^. Hu et al. have obtained mono layer nanosheet of NiFe LDH and thus exposed more active sites^11^. Yang et al. studied the synergistic interactions between doping atom Cr and NiFe LDH in the NiFeCr LDH electrocatalyst, which enhanced the OER activity in the alkaline solutions^32^.

However, the powdered electrocatalysts are often applied with some insulating polymer, resulting in low conductivity and poor catalytic performance^33^. In addition, the stability of powdered active materials is often adversely affected due to weak physical binding force between the substrate and electrocatalysts^34^. Compared to traditional electrode, 3D electrodes show more efficient charge transport and better stability. 3D electrodes are usually selected for high surface area, high conductivity, and easy binding to active substances, for example, carbon paper^35^, nickel foam^36^, carbon cloth ^37^ and so on. Among them, stainless steel is the ideal 3D electrodes due to their excellent conductivity and robustness. Compared to other common 3D electrodes, stainless steel contains rich transition metal (TM) elements, like Fe, Ni, Cr et al., which are active sites in many electrochemical reactions^38^. In situ or ex situ surface modification is the main method to design stainless steel electrode. Schäfer et al. have reported various types of stainless steel was modified the surface by in situ or ex situ oxidation and corrosion, and thus improved the performance in the water splitting^39,40^. Balogun et al., Lyu et al., and Liu et al. doped the heteroatoms on the surface of stainless steel through nitriding, phosphating, sulfuration and carbonization, so as to introduce more active sites and enhance the efficiency of electrochemical water splitting^41–43^. However, the surface modification mentioned above refers to high temperature or vacuum process, complex equipment, which means too high production cost and difficult to repeat process, and is not beneficial to industrial application.

In this work, a facile and rapid route is reported to fabricate the Cr doped FeNi LDH nanosheet with ultra small scaled on situ modified stainless steel under ambient condition. Especially, the modified SS electrocatalysts exhibit outstanding OER activity, which achieves a Tafel slope of 44 mV dec^−1^ and over potential of 280 mV at 10 mA cm^−2^. The existence of ultra-small NiFe LDH nanosheets and strong adhesion between FeNi LDH nanosheets and SS substrate promote excellent stability for the prepared electrolytes. To sum up, this work provides a cost-effective and facile strategy to optimize both the structure and composition of the SS-based electrode and design efficient active and durable electrocatalysts for water splitting.

## Methods

### Sample preparation

#### Preparation of etched SS (ESS)

The commercial 316L type SS mesh (2.0 cm × 4.0 cm, Tianhong Stainless Steel Co., Ltd) were cleaned ultrasonically in distilled water and ethanol for 15 min, respectively. After cleaning process, the cleaned SS were etched ultrasonically in 6 M HCl for 120 min. Then, the etched SS were washed with distilled water thoroughly to remove the residual acid and other impurity, and then dried in 24 h at 60 ℃ in oven. The obtained samples were denoted as ESS.

#### Preparation of Cr doped FeNi LDH/ ESS (Cr–FeNi LDH @ ESS)

Firstly, 120 mL of the NaOH (12.8 g) solution and 40 mL of the (NH_4_)_2_S_2_O_8_ (1.2 g) were mixed in a 250 mL breaker under stirring. Then several pieces of prepared ESS were immersed into the mixed solution mentioned above at different temperature (25 ℃, 50 ℃, 80 ℃) for 60 min. After reaction, the samples were washed with deionized water for several times. Finally, the samples were dried in a 60 ℃ oven to obtained the Cr–FeNi LDH @ ESS.

### Sample characterization

The morphology and microstructure of samples were investigated by field emission scanning electron microscope (FESEM, Hitachi, S4800). To further determine the microstructure and chemical composition of samples, high resolution transmission electron microscopy (HRTEM, Themis) with mapping scanning energy dispersive X-ray spectroscopy (EDS) was employed. Before HRTEM test, the surface of samples were subjected strong ultrasonic peeling in ethyl alcohol for 1 h, so that the nanosheet on the surface of samples could be disperse in the ethyl alcohol, and then droped casting on the Cu-microgate. The microgate sample mentioned above was analyzed by HRTEM. The X-ray photoelectron spectroscopy (XPS) technology performed on an ESCALAB 250Xi X-ray photoelectron spectrometer using Mg as the excitation source was employed to detect the chemical state of the elements on the surface of the samples. All binding energies were referenced to the C 1 s peak (284.8 eV) arising from adventitious carbon. The crystalline structure of samples were investigated by 2θ X-ray diffraction (XRD) using a Rigaku diffractometer (Rigaku Ultima IV) with the grazing angle of 1° at the scan rate of 2° min^−1^. The Raman spectra were obtained on an InVia Raman microscope (Renishaw, England) in backscattering geometry with a CCD detector.

### Electrochemical measurements

An electrochemical workstation (CHI 760E, CH instrument) with a three electrode cell, using carbon rod as counter electrode and saturated calomel electrode (SCE) as reference electrode, was employed to processing all the electrochemical measurements in 1 M KOH electrolyte. The working electrode was as-prepared sample mentioned above. The linear sweep voltammetry (LSV) was performed at a scan rate of 5 mV s^−1^. Potentials were calibrated to a reversible hydrogen electrode (RHE) based on the equation: E_*RHE*_ = E_*SCE*_ + 1.05 V. To explore the electrode chemically active surface (C_*dl*_), the CV measurements of working electrodes is carried out for two cycles between 1.09 and 1.25 V vs. RHE, and the scan rate was set to 20, 40, 60, 80 and 100 mV, respectively. The C_*dl*_ is estimated from the linear slope of the current density (*ΔJ*) against the scan rate. The electrochemical impedance spectroscopy (EIS) was analyzed by an Autoab electrochemical workstation (Autolab PGSTAT302N, MetrohmAutolab BV, Netherlands) at a potential of 1.55 V vs. RHE.

## Results and discussion

### Materials characterization

**Figure 1:**
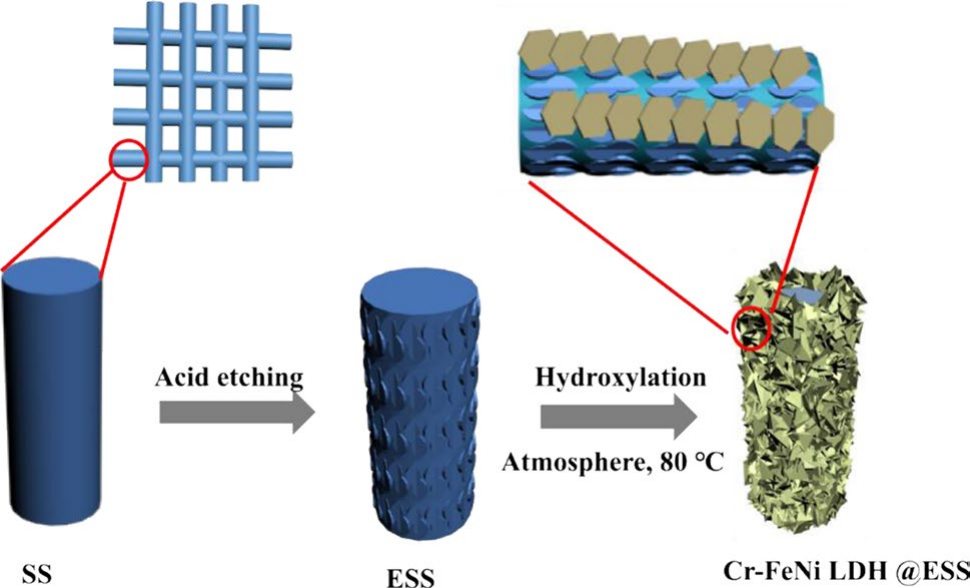
Schematic illustration of the fabrication procedure of Cr–FeNi LDH @ ESS electrodes.

**Figure 2:**
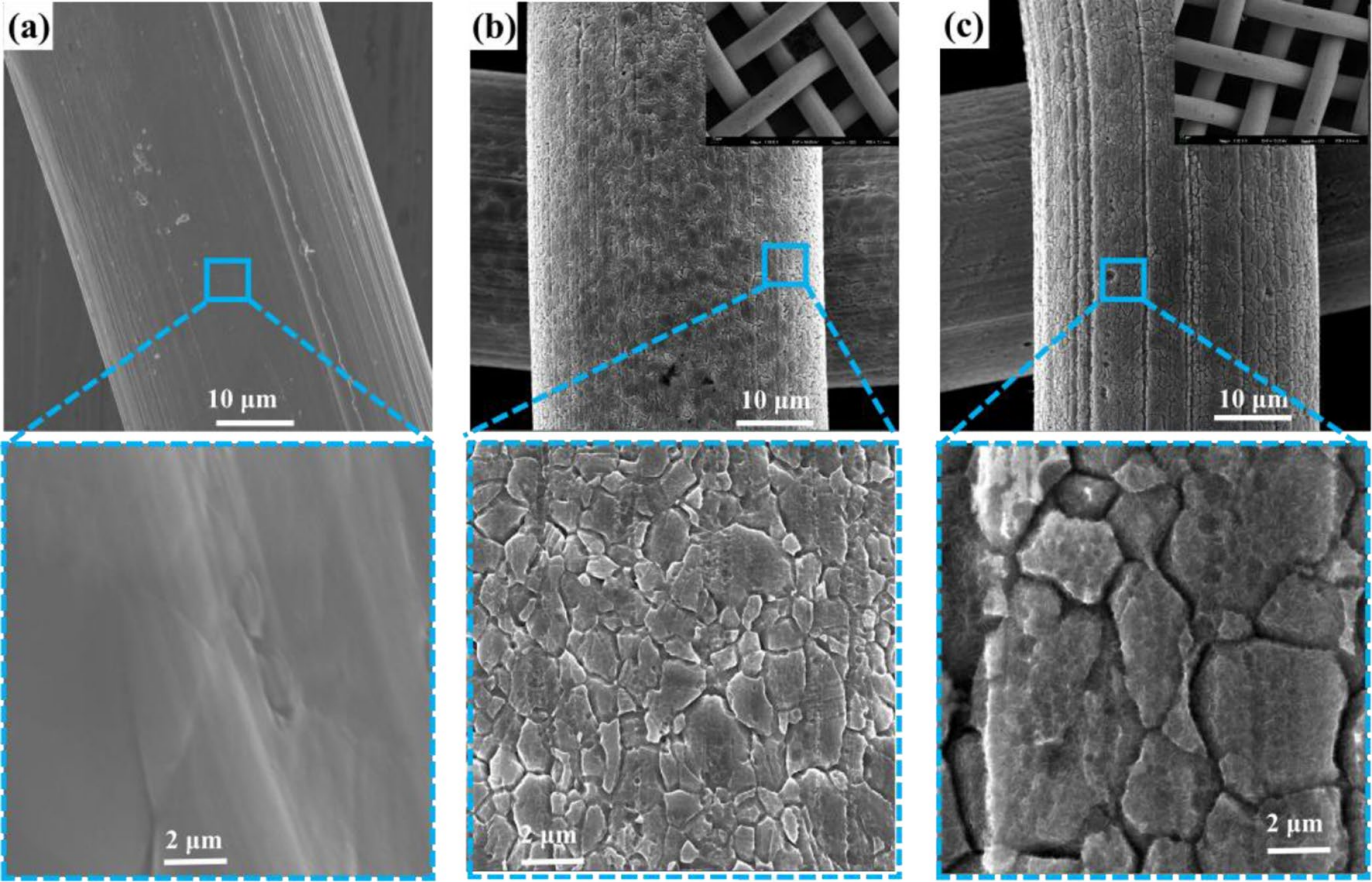
FESEM images of surface morphology about (**a**) SS, (**b**) ESS and (**c**) Cr–FeNi LDH @ ESS samples in low and high magnification.

As illustrated in Fig. 1, the electrodes of Cr–FeNi LDH @ ESS were fabricated by only simple two steps, including acid etching and wet chemical hydroxylation in atmosphere condition. In this work, the Cr–FeNi LDH were in-situ synthesized on ESS mesh. The surface morphology of ESS based samples were investigated by scanning electron microscopy (SEM). As shown in Fig. 2a–c, the surface morphology of the SS mesh has changed obviously after various treatments mentioned above, but the 3D network has not been damaged. The SEM results show that the surface of ESS exhibits gully erosion microstructure, which provide the template for the growth of Cr–FeNi LDH. After wet chemical reaction, 2D nanosheets were tightly packed on the surface of ESS, as shown in Fig. 2c.

**Figure 3:**
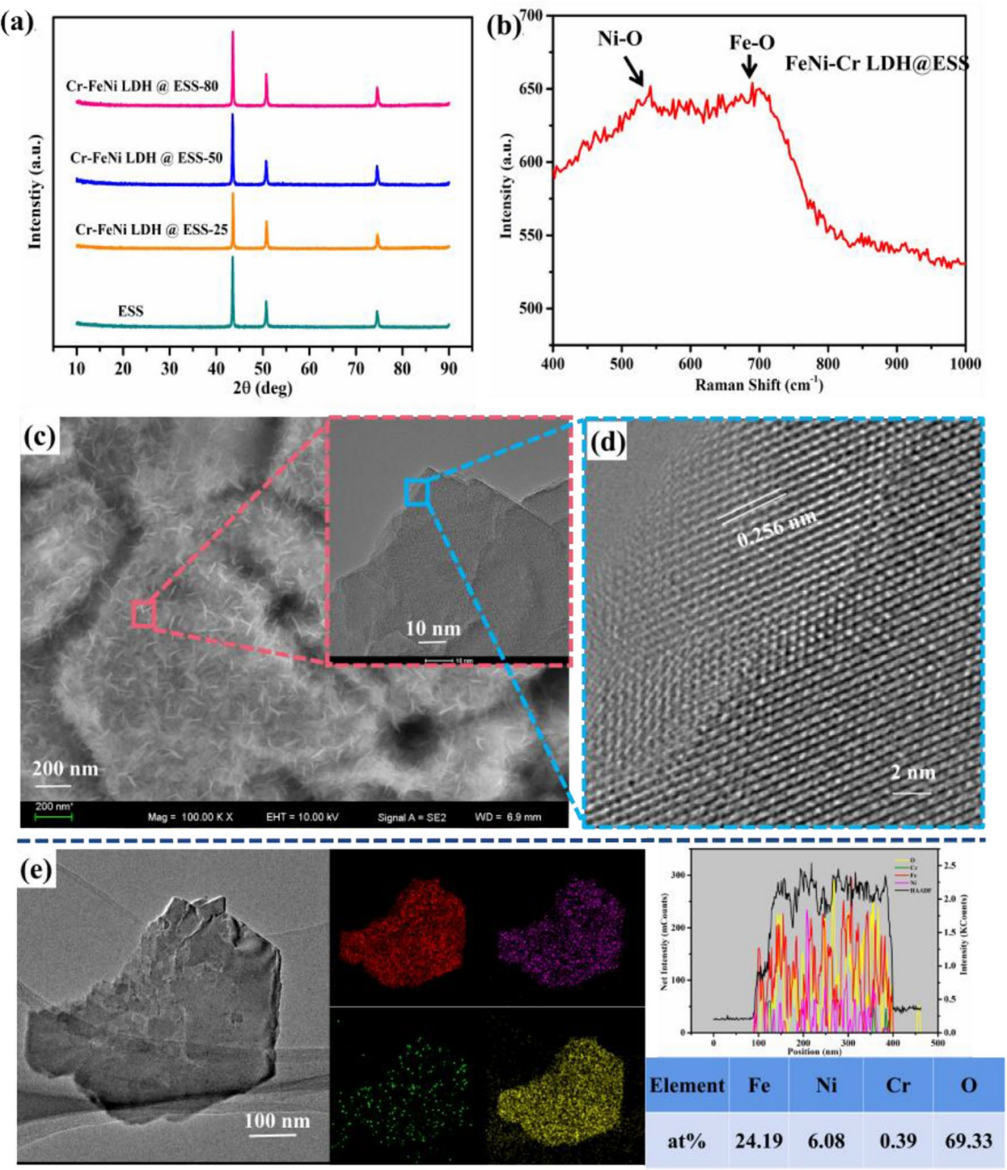
(**a**) XRD datas of ESS sample and Cr–FeNi LDH @ESS samples fabricated at different temperature; (**b**) Raman spectrum of Cr–FeNi LDH @ESS. (**c**) FESEM image of Cr–FeNi LDH @ESS and TEM image shown in the inset; (**d**) HRTEM image of Cr–FeNi LDH @ESS; (**e**) the elements mapping and compositions of Fe, Ni, Cr and O.

The microstructure of ultra small size nanosheets for electrodes indicates high specific surface area and rich exposed edges with lots of active sites. According to the X-ray diffraction (XRD) pattern (shown in Fig. 3a), it can be seen all the ESS based samples had the same three peaks near the 43.6°, 50.7°, and 74.7°, which agree well with that for the austenite phase (PDF card #33-0397). It is noted that the as-prepared Cr–FeNi LDH @ ESS is too small to be detected by XRD, so Raman technology was employed to determine the formation of the compoud on the surface of ESS. As shown in Fig. 3b, the Raman peaks located at 546.4 and 670.7 cm^−1^ under 532 nm excitation are allocated to the vibration of Ni–O and Fe–O bond^44^. High resolution transmission electron microscope (HRTEM) was used to observe the structure of the samples. FESEM and TEM magnification image (Fig. 3c) shows that the nanosheet is uniform and have ultra tiny scale with a diameter of about 20 nm, which can lead to highly exposed active edge sites and catalytic activity. The lattice fringe shown in Fig. 3d is 0.256 nm, which is in agreement with the (012) plane of the FeNi LDH crystal. The EDS mappings of Cr–FeNi LDH @ ESS indicate the homogeneous distribution of Fe, Ni, O and Cr on the nanosheets, while the Cr signal is extremely weak, indicating only a few Cr atoms doped in the FeNi LDHs. It is observed in Fig. 3e that the ratio of Fe/Ni is close to 4, far from the original elementary composition of SS, indicating the Fe and Cr elements were run off after undergoing acid treatment and hydroxylation reaction. Base on the result above, it suggests that the Cr–FeNi LDHs were successfully in-situ fabricated on the ESS mesh.

**Figure 4:**
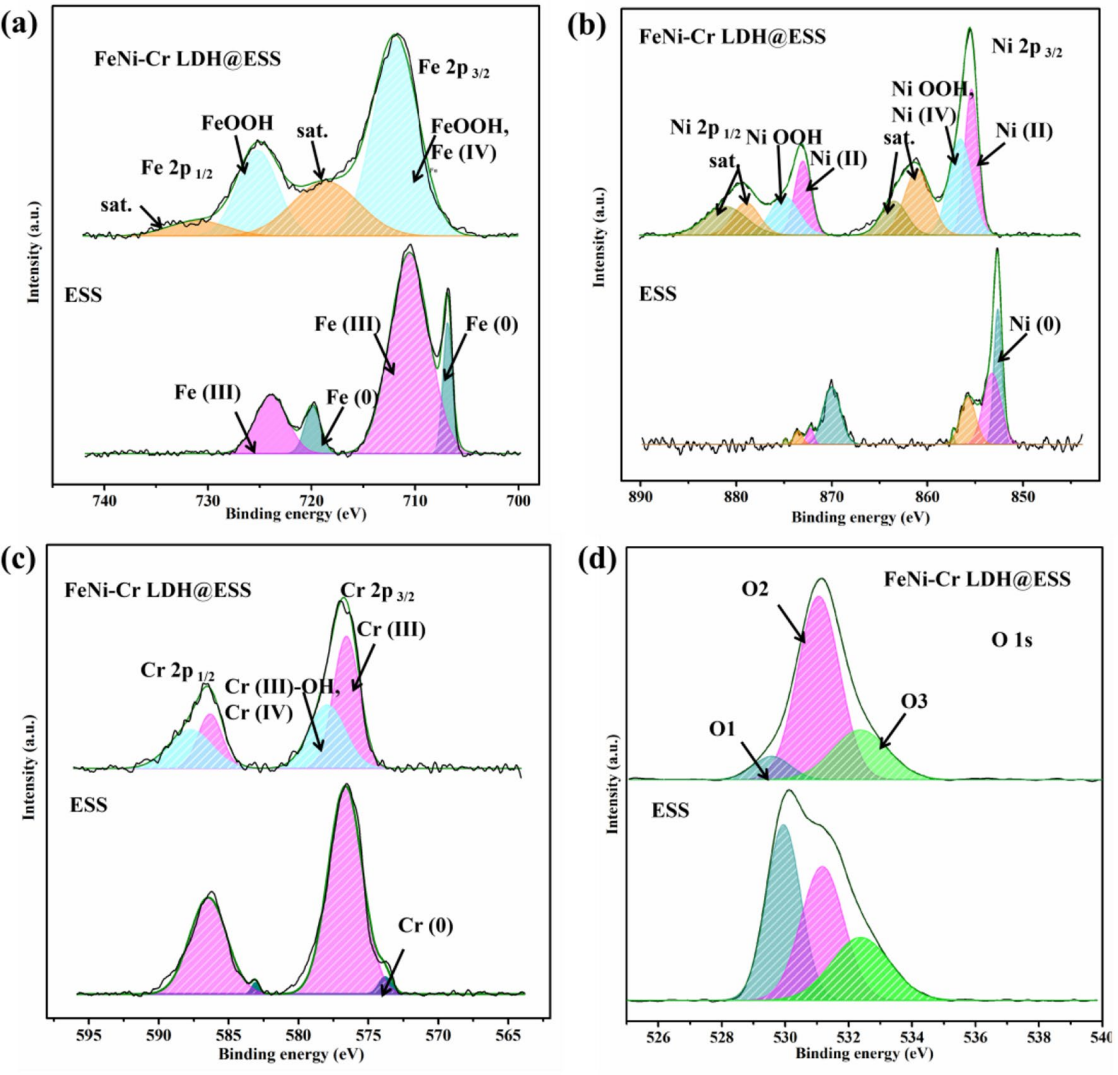
XPS data of ESS and Cr–FeNi LDH @ ESS sample. (**a**) Fe 2p 3/2, (**b**) Ni 2p 3/2, (**c**) Cr 2p 3/2 and (**d**) O 1 s.

The surface active species were further confirmed by XPS. Consistent with EDS results, the XPS spectra showed Fe, Ni, Cr and O elements exist on the surface of samples (Fig. 4). The O1s spectra (shown in Fig. 4d) can be split into three peaks at 529.7, 531.1 and 532.3 eV, which associated with meta-oxygen (labeled as O1), hydroxyl group (OH, labeled as O2), and adventitious carbon oxygen species or adsorbed water molecules (labeled as O3), respectively^45–47^. The *OH and*OOH are intermediate during four electrons OER reaction, so their existence can speed up the reaction and regarded as active species in the OER reaction. It reveals that the surface of ESS is oxidized due to the existence of metal oxidation state. The binding energies of Fe 2p3/2 and 2p1/2 peak for ESS sample can be split into two peaks located at 706.8 eV, 710.5 eV, 719.8 eV and 723.8 eV, respectively, which are assigned to elementary iron (Fe) and Fe (III) ^48,49^. Similarly to Fe spectra, both of Ni and Cr peak can be deconvoluted into two peaks involving chemical state zero-valent metal and metal oxide, shown in Fig. 4a–c. Compare to the ESS sample, all the peaks of metal elements for the Cr–FeNi LDH @ ESS sample move towards higher binding energy, suggesting that highly oxidation state of metal formed on the surface and the metal ions were in the electron-deficient state. As shown in the Fig. 4a, the Fe spectra with Fe 2p3/2 and 2p1/2 peak of Cr–FeNi LDH @ ESS sample centered at 711.8 eV and 725.2 eV, respectively, indicating that there are overlapped chemical state including Fe (IV) and Fe (III) bonded to *OOH^50^. According to deconvoluted peak of the Ni and Cr spectra, the binding energy located at 856.6 eV and 578.8 eV belongs to multiple chemical states containing of Ni (IV), Cr (IV), Ni (III) and Cr (III) bonded to hydroxy, respectively^51,52^. It is reported that the redox- active cations with high oxidation states (such as Fe^3+^, Fe^4+^, Ni^3+^, Ni^4+^, Cr^3+^, Cr^4+^) serve as effective active sites and buffer the multi electron process for water oxidation^32^. Meanwhile, the metal hydroxide matrix has a positive synergistic roles between redox-active cations (Ni, Fe and Cr) and Lewis-acid cations (Fe and Cr)^32^.

The XPS result further confirmed the formation of Cr–FeNi LDH on the surface Cr–FeNi LDH @ ESS sample (Fig. 4c). After hydroxylation reaction, the Cr is highly oxidized to an electron-deficient state and bonded active group (*OH, shown in Fig. 4c), indicating Cr atoms were doped in the FeNi LDH compound and have a supporting role in the observed activity.

### Electrocatalytic performance

**Figure 5:**
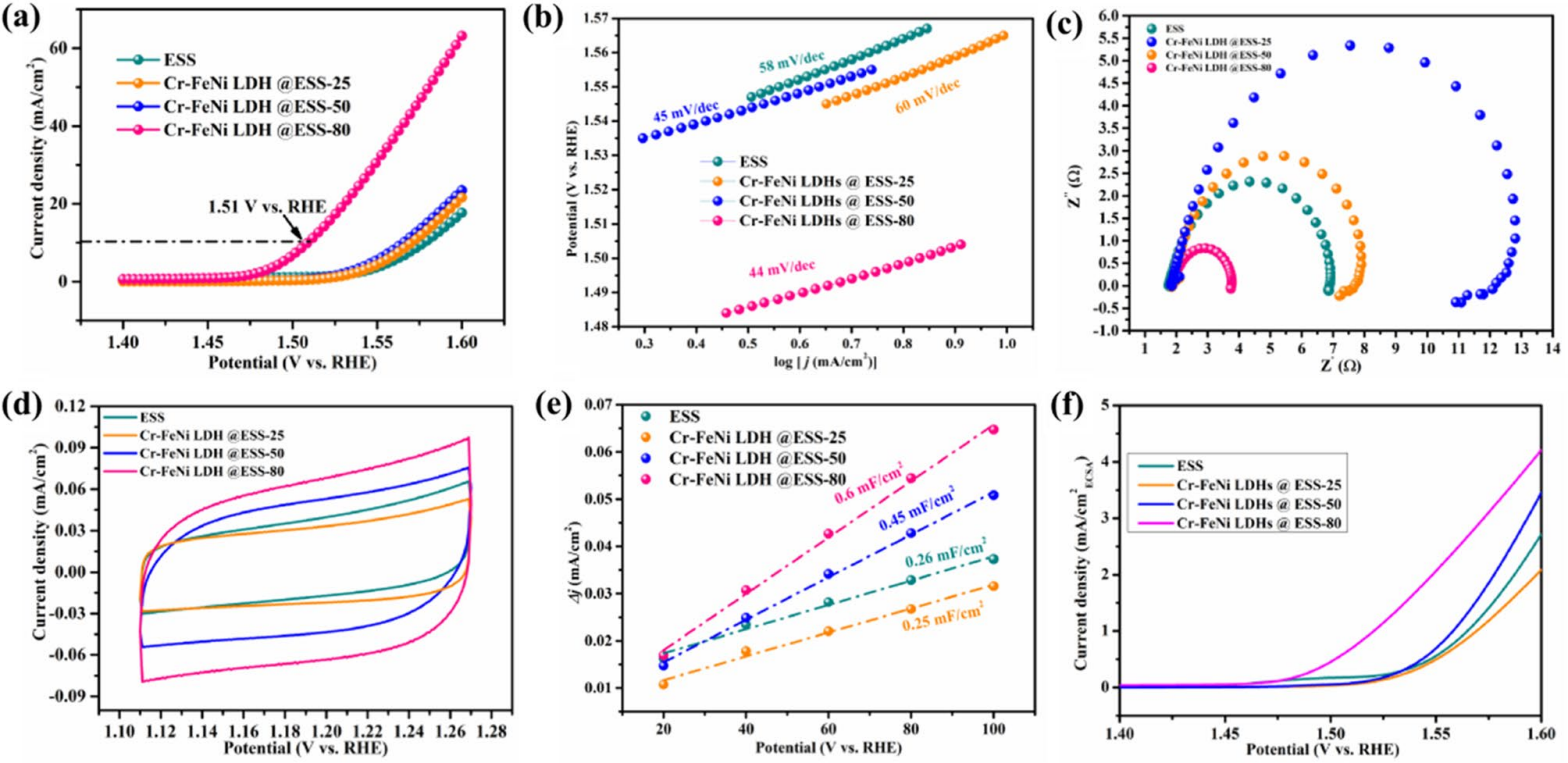
The electrocatalytic activity of ESS samples and Cr–FeNi LDH @ ESS samples fabricated at different temperature. (**a**) LSV curves, (**b**) corresponding Tafel plots of the samples, (**c**) EIS curves of the samples at the potential of 1.55 V, (**d**) CV curves of the samples at the scan rate of 100 mV s^−1^, (**e**) C_dl_ values of samples determined by the slope of a line formed from capacitive current at 1.19 V vs. RHE under various scan rates, and (**f**) the OER performance of samples after the electrochemical active area (ECSA) normalization.

The electrocatalytic activity of all the samples was assessed with three-electrode system in 1 M KOH electrolyte. As shown in Fig. 5a, the Cr–FeNi LDH @ ESS electrode showed better OER performance than ESS sample, which needs only a low overpotential of 280 mV to reach a current density of 10 mA cm^−2^. To further optimize the catalytic properties of prepared electrode, the different fabricated parameters was investigated for Cr–FeNi LDH @ ESS samples, which were prepared at different temperatures (25 ℃, 50 ℃, 80 ℃). As shown in Fig. 5a. the Cr–FeNi LDH @ ESS sample treated at relatively low temperature exhibit poor OER properties, due to the stable anti-oxygenic and anti-corrosion resistance of SS in mild condition. This result implied SS-supported electrode would get long stability in normal electrolyte and atmospheric temperature environment. To estimate the electrocatalytic kinetics, the Tafel slope is list in Fig. 5b. Compared to ESS samples, the Cr–FeNi LDH @ ESS sample possesses lower Tafel slope value with 44 mV dec^−1^, indicating the Cr–FeNi LDH @ ESS sample possesses faster catalytic kinetic. The electrocatalytic kinetics of OER was also analyzed by electrochemical impedance spectroscopy (EIS) technology. In this work, the charge transfer resistance at 1.55 V on all the samples have been estimated. As shown in Fig. 5c, the Cr–FeNi LDH @ ESS sample has smaller diameter of Nyquist plot in the EIS test than ESS samples, which demonstrate a fast electron transfer for OER and hence the optimized electrocatalytic OER activity was realized. The layered Cr–FeNi LDH has favorable charge transfer resulting from the redox reactions with multivalent metal cations in the layers and intercalated anions migrate within the interlayer space^53,54^.

The CV curve integral area can represent active specific surface area (A_s_), as shown in Fig. 5d, the A_s_ of Cr–FeNi LDH @ ESS is much larger than ESS samples. To further assess the electrochemically active surface area of Cr–FeNi LDH @ ESS and other samples, the electrochemical double-layer capacitance (C_dl_) was evaluated (shown in Fig. 5e). It reveals that the C_dl_ of Cr–FeNi LDH @ ESS (0.6 mF cm^−2^) is over six times higher than bare ESS mesh. It is observed the hydroxylation reaction is more complete with higher reaction temperature, so that the surface activity increased. The electrochemically active surface area (ECSA) was calculated based on the previous report^4^. Then, after the ECSA normalization (Fig. 5f), the specific activity of Cr–FeNi LDH @ ESS is higher than other electrodes, indicating the advanced catalytic activity of the Cr–FeNi LDH @ ESS.

In addition, many reports elaborated the Cr elements was a catalytically inactive specie, which should be removed, migrated, displaced or transformed on the top layer of the stainless steel during surface modification. However, Cr is important component of stainless steel, which is indispensable for current conduction and corrosion resistance. The OER performance of Cr–FeNi LDH @ ESS in this work is superior to some reported work for Cr based LDHs catalysts, as shown in Table 1. Therefore, we kept a few Cr content on the surface of Cr–FeNi LDH @ ESS samples after surface modification, and thus make sure the long time stability of the electrodes in the electrolyte.

**Table 1:** OER activity of Cr based catalysts.

Cr based catalysts	Overpotential (mV)	Tafel slope (mV/dec)	Electrolyte	References
Cr–FeNi LDH @ ESS	280 @ 10 mA/cm^2^	44	1 M KOH	In this work
NiFeCr LDH/Carbon paper	280 @ 10 mA/cm^2^	69	1 M KOH	^55^
γ-CrooH/Ni foam	334 @ 50 mA/cm^2^	41	1 M KOH	^56^
NiFeCr LDH-MoS_2_	270 @ 10 mA/cm^2^	85	1 M KOH	^57^
Cr^6+^ @ graphene	197 @ 10 mA/cm^2^	–	1 M KOH	^58^
Co_2_Cr LDH	340 @ 10 mA/cm^2^	87	0.1 M KOH	^59^

**Figure 6:**
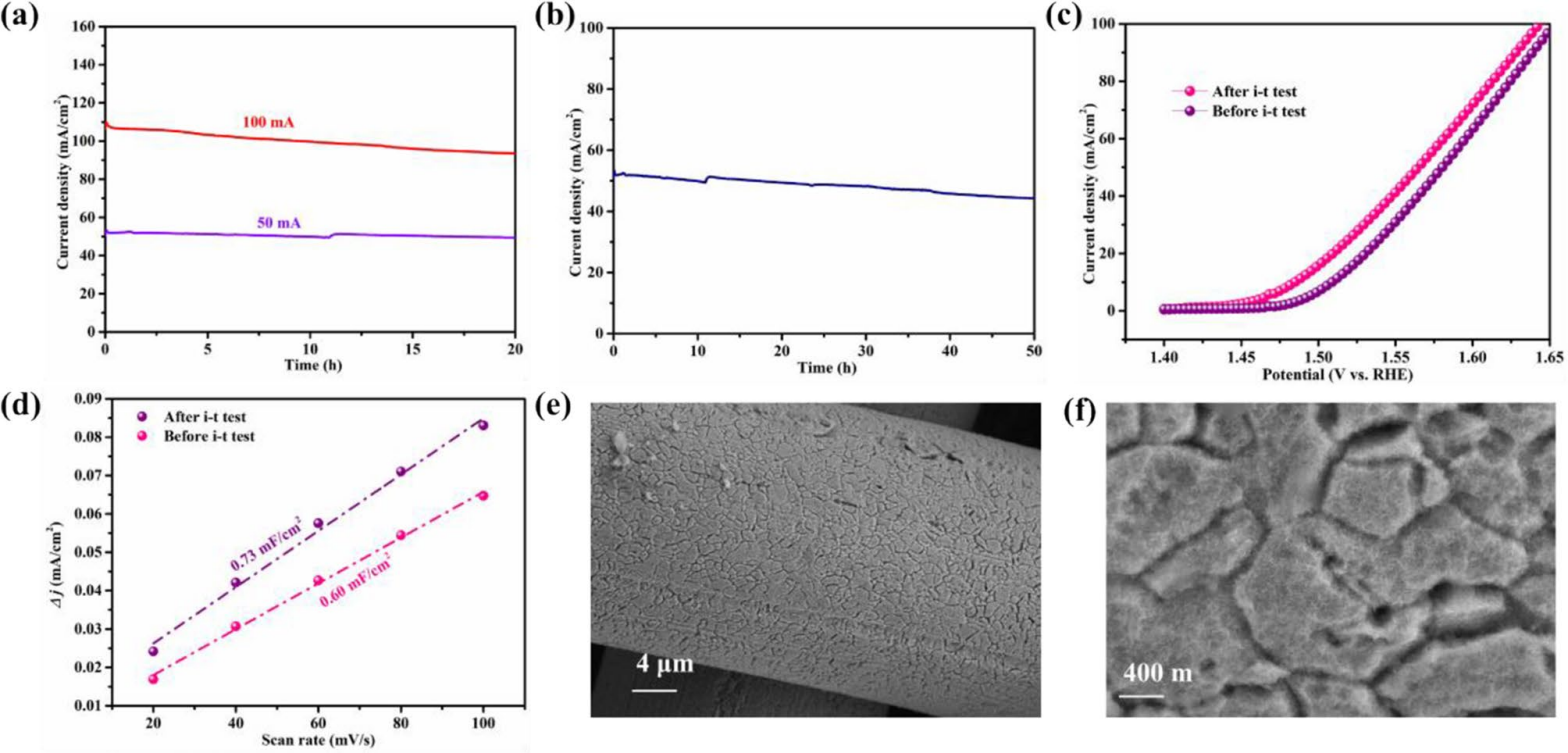
(**a**) Time-dependent current curves of Cr–FeNi LDH @ ESS sample under a static potential of 1.56 V vs. RHE and 1.66 V vs. RHE for 20 h. (**b**) Time-dependent current curves of Cr–FeNi LDH @ ESS sample under a static potential of 1.56 V vs. RHE for 50 h, (**c**) LSV curves and (**d**) C_dl_ of Cr–FeNi LDH @ ESS sample before and after 50 h electrolysis. FESEM images of surface morphology about Cr–FeNi LDH @ ESS sample after 50 h electrolysis in low (**e**) and (**f**) high magnification.

To evaluate the stability of the electrocatalysts, long term i-t test of Cr–FeNi LDH @ ESS were carried out at a constant potential of 1.56 V and 1.65 V vs. RHE for 20 h. As shown in Fig. 6a, the initial current of samples is around 50 mA cm^−2^ and 110 mA cm^−2^ in 1 M KOH electrolyte, respectively. After 20 h test, the current density of Cr–FeNi LDH @ ESS sample revealed negligible change at potential of 1.56 V vs. RHE, and slight reduction at 1.65 V vs. RHE.

After long term i-t test of around 50 mA cm^−2^ (shown in Fig. 6b), the catalytic activity of Cr–FeNi LDH @ ESS for electrocatalytic OER was further estimated in 1 M KOH. As shown in Fig. 6c, the oxygen evolution overpotential after i-t test of Cr–FeNi LDH @ ESS moved toward lower potential than the original sample, indicating the surface of Cr–FeNi LDH @ ESS sample was activated after i-t test for 50 h. The electrochemical double-layer capacitance (C_dl_) for sample after i-t test was calculated in Fig. 6d, which is increased to 0.73 mF cm^−2^ higher than original one. It is implied more active sites were generated after i-t test. In addition, due to the good chemical stability of ESS mesh, the appearance of the samples has not changed after the long-time stability test, as shown in Fig. 6e,f. Thus, the Cr–FeNi LDH @ ESS sample exhibit excellent stability for OER.

**Figure 7:**
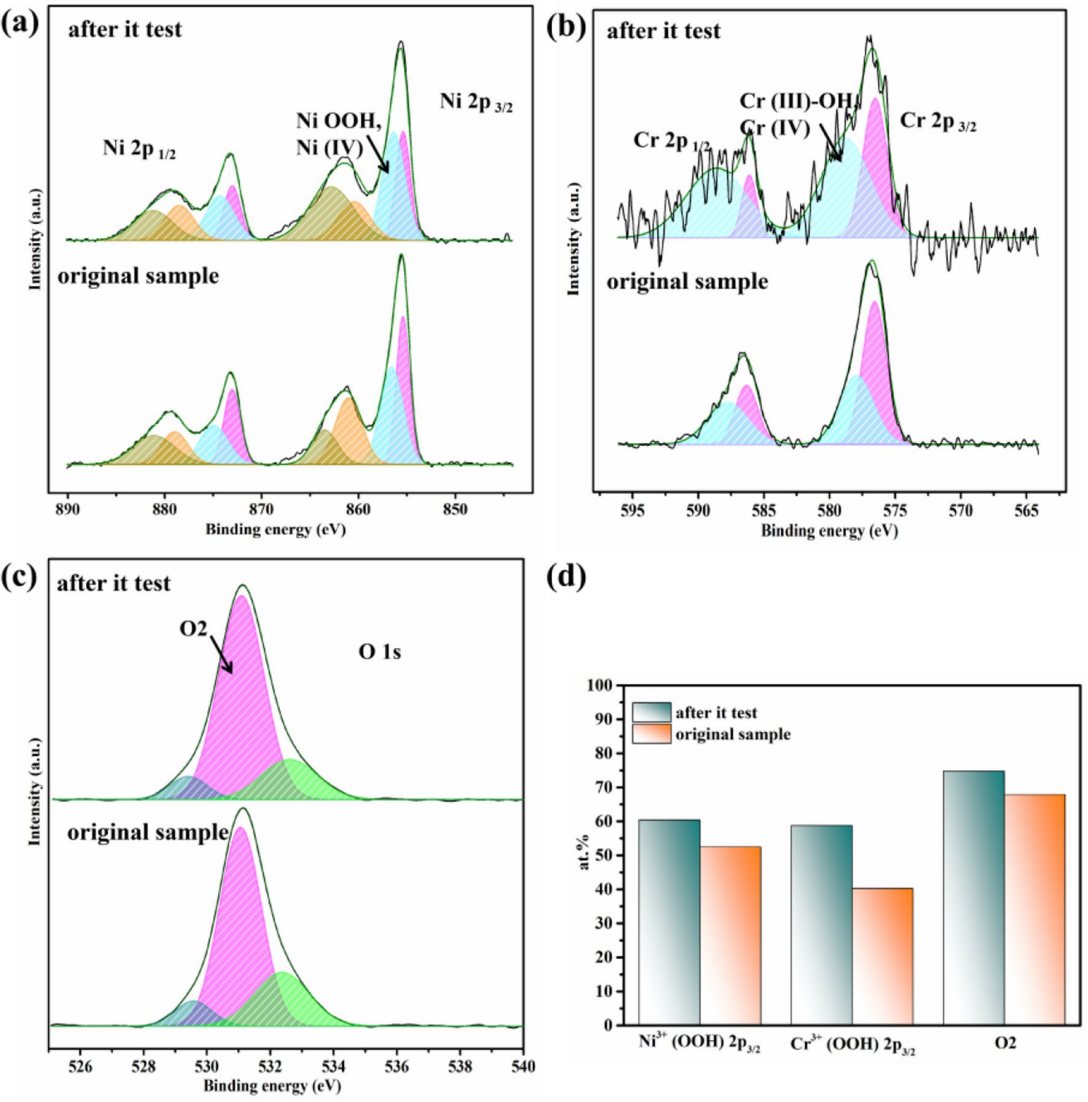
XPS data of ESS and Cr–FeNi LDH @ ESS sample after i-t test. (**a**) Ni 2p 3/2, (**b**) Cr 2p 3/2, (**c**) O 1 s and (**d**) the atomic ratio of high multivalent oxidation state and oxyhydroxide for Ni and Cr elements, as well as O_2_, respectively.

To further verify the active species on the surface of the samples, the XPS was applied to evaluate the Cr–FeNi LDH @ ESS before and after i-t tests. As displayed in the Fig. 7, it revealed that the content of high multivalent oxidation state and oxyhydroxide for Ni and Cr elements were increased about 10% and 20%, respectively. Besides, the hydroxy (O2) was also got a growth of 5%. It indicates active species like oxyhydroxides were generated during the oxygen evolution, and thus further improve the catalytic activity.

## Conclusion

In summary, a facile and rapid route was used to in situ fabricate the Cr–FeNi LDH, on modified stainless steel under ambient condition. The prepared Cr–FeNi LDH @ ESS samples exhibit excellent electrocatalytic performance for OER, with a low overpotential of 280 mV at the current density of 10 mA cm^−2^ and an outstanding kinetics with the Tafel slope of 44 mV dec^−1^. The exceptional electrocatalytic properties mainly results from the formation of the unique ultra small 2D structures of Cr–FeNi LDH, metal ions with multivalent oxidation state (such as Fe^3+^, Fe^4+^, Ni^3+^, Ni^4+^, Cr^3+^, and Cr^4+^), which promote the exposure of active sites and thus increase the electrocatalytic activity. Furthermore, the strategy of in-situ growth and intrinsic corrosion resistance of stainless steel enhance the stability of the self-supported Cr–FeNi LDH @ ESS electrodes in the 1 M KOH electrolyte. This work provides a green, simple and low-cost strategy to design highly efficient and durable electrocatalyst for water splitting.

## Data Availability

The datasets used and/or analysed during the current study available from the corresponding author on reasonable request.
